# *Aureobasidium pullulans* volatilome identified by a novel, quantitative approach employing SPME-GC-MS, suppressed *Botrytis cinerea* and *Alternaria alternata in vitro*

**DOI:** 10.1038/s41598-020-61471-8

**Published:** 2020-03-11

**Authors:** S. M. Yalage Don, L. M. Schmidtke, J. M. Gambetta, C. C. Steel

**Affiliations:** School of Agricultural and Wine Sciences, National Wine and Grape Industry Centre, Charles Sturt University, Locked Bag 588, Wagga Wagga, New South Wales 2678 Australia

**Keywords:** Metabolomics, Antifungal agents, Applied microbiology, Fungal pathogenesis, Pathogens

## Abstract

Volatile organic compounds (VOCs) produced by *Aureobasidium pullulans* were investigated for antagonistic actions against *Alternaria alternata* and *Botrytis cinerea*. Conidia germination and colony growth of these two phytopathogens were suppressed by *A. pullulans* VOCs. A novel experimental setup was devised to directly extract VOCs using solid-phase microextraction-gas chromatography-mass spectrometry (SPME-GC-MS) from antagonist-pathogen culture headspace. The proposed system is a robust method to quantify microbial VOCs using an internal standard. Multivariate curve resolution-alternating least squares deconvolution of SPME-GC-MS spectra identified fourteen *A. pullulans* VOCs. 3-Methyl-1-hexanol, acetone, 2-heptanone, ethyl butyrate, 3-methylbutyl acetate and 2-methylpropyl acetate were newly identified in *A. pullulans* headspace. Partial least squares discriminant analysis models with variable importance in projection and selectivity ratio identified four VOCs (ethanol, 2-methyl-1-propanol, 3-methyl-1-butanol and 2-phenylethanol), with high explanatory power for discrimination between *A. pullulans* and pathogen. The antifungal activity and synergistic interactions of the four VOCs were evaluated using a Box-Behnken design with response surface modelling. Ethanol and 2-phenylethanol are the key inhibitory *A. pullulans* VOCs against both *B. cinerea* and *A. alternata*. Our findings introduce a novel, robust, quantitative approach for microbial VOCs analyses and give insights into the potential use of *A. pullulans* VOCs to control *B. cinerea* and *A. alternata*.

## Introduction

Microbial antagonists have been widely explored as more environmentally friendly disease management alternatives to reduce the excessive use of synthetic fungicides^1,2^. Among a wide array of mechanisms exploited by microbial antagonists, the production of volatile organic compounds (VOCs) with antimicrobial properties^3^ has become popular in the recent research due to their low risk of toxic residues, biodegradability^4^ and enormous spectrum of activity regardless of a physical contact of the commodity^5^.

*Aureobasidium pullulans* is a yeast-like saprophytic fungus, naturally inhabiting plant and fruit surfaces^6^. This yeast is a well-known biocontrol agent against a range of pathogenic fungi^7,8^ and strains of the organism have been formulated as commercial preparations for the practical management of fungal phytopathogens of a range of horticultural crops^9^. Production of antifungal VOCs has been recently identified as a potential mode of action for its biocontrol^10,11^. One of these studies has confirmed the identity of its four VOCs, 2-methyl-1-butanol, 3-methyl-1-butanol, 2-methyl-1-propanol and 2-phenylethanol^11^. These compounds were shown to suppress conidia germination and mycelium growth of *Botrytis cinerea*, *Colletotrichum acutatum*, *Penicillium expansum*, *Penicillium digitatum* and *Penicillium italicum* under both *in vitro* and *in vivo* conditions^11^. A similar study identified over 20 VOCs from *A. pullulans* isolated from grapevine^12^. However, none of these compounds were tested for antifungal properties^12^. Thus, further investigations are necessary to identify the antifungal properties of *A. pullulans* VOCs. In the absence of this knowledge, it is difficult to fully understand the mode of action of *A. pullulans* as a biological control agent. Four *A. pullulans* isolates were used in the current study. All four isolates have been previously proven to have antifungal properties against *Greeneria uvicola* in a dual culture system which reduced the radial growth of the pathogen compared to controls^8^. However their ability to produce VOCs has not been investigated.

The present study investigated *A. pullulans* antifungal VOCs against two necrotrophic fungal pathogens; *Botrytis cinerea* and *Alternaria alternata*. The two pathogens infect a number of horticultural crops causing both pre and postharvest yield losses worldwide^13,14^. Black mould and grey mould are two plant diseases attributed to *A. alternata* and *B. cinerea* respectively^13,14^. In addition to yield losses, the production of deleterious off flavours leads to a loss of wine quality when wine is made with grey mould infected grapes^15^.

To our knowledge, a robust automated technique for quantitative analysis of microbial VOCs has not previously been reported to assess antagonist and pathogen interaction and the inhibitory actions of VOCs. We propose a novel set up for the quantitative analysis of VOCs by automated solid phase-microextraction-gas chromatography-mass spectrometry (SPME-GC-MS). The setup facilitates extraction of VOCs from the headspace of *A. pullulans* and pathogen interaction system. Such a system allows variations in VOCs production by antagonists and the influence of target pathogens to be monitored. Previous studies manually extracted VOCs from a double Petri dish system^11,16^ without incorporation of suitable internal standards (IS). Thus, studies on the efficacy of mixtures of microbial VOCs have based their findings on the proportions of relative peak areas, derived from GC-MS chromatograms^17,18^. However, the degradation of SPME fibre sensitivity between injections, different affinities of different analytes to SPME fibres^19^ and detector sensitivity to different analytes^11^ make the interpretation of volatile profiles challenging. The employment of appropriate ISs for VOCs peak area normalisation generally overcomes these constraints and is widely practised in areas such as wine and food metabolomics^20^. The current study proposes a method to introduce an IS to the antagonist-pathogen culture system without disturbing the headspace and thereby perform quantitative sampling of VOCs.

Multivariate chemometrics which uses a combination of mathematical and statistical approaches^21^, have become important in data resolution obtained from analytical platforms such as gas chromatography-mass spectrometry (GC-MS)^22^. A range of unsupervised and supervised approaches to resolve complex multivariate data sets allows identification of compositional differences between samples^22^. Multivariate Curve Resolution-Alternating Least Squares (MCR-ALS), one of the popular chemometric methods used in the resolution of multiple component responses^23^, was employed in this study in an association with untargeted, SPME-GC-MS analysis, to characterise *A. pullulans* VOCs.

The antifungal activity of VOCs has been suggested to be synergistic or additive rather than a sole inhibitory action^11,24^. An inability to mimic the antagonists’ antimicrobial activity by VOCs when applied individually^24^ and production of VOCs at very low concentrations by antagonists^25^ suggest that a synergistic or additive effect may occur. In this context, our study aimed to identify *A. pullulans* VOCs by a robust, direct, quantitative approach based on untargeted metabolomics and chemometric analysis. Also the antifungal properties of VOCs were evaluated against *B. cinerea* and *A. alternata*. Lastly, the synergistic behavior of VOCs to cause antifungal effects were studied using a response surface modelling (RSM) approach.

## Methods

### Antagonist and pathogen isolates

*A. pullulans* (KR605651.1(A1), KR605650.1(A2), KR605653.1(A3), KR605665.1(A4), *B. cinerea* TN080 (all isolated from wine grapes)^8^ and *B. cinerea* DAR69764 (isolated from tomato) were obtained from the culture collection of the laboratory of National Wine and Grape Industry Centre, Charles Sturt University, Wagga Wagga, Australia. *A. alternata* (MH931372) was isolated from a tomato fruit showing characteristic black mould symptoms. Cultures were maintained on Potato Dextrose Agar (PDA) at 25 °C for routine use.

### Evaluation of the antifungal activity of *A. pullulans* VOCs

#### Mycelium growth inhibition

A double Petri dish method^26^ was adopted to evaluate the effect of VOCs on mycelium growth. A cell suspension of *A. pullulans* (10^8^ cells/mL, 100 µL), harvested in sterile distilled water (SDW), was spread on PDA, sealed and incubated at 25 °C for 48 h. Mycelial agar discs (7 mm diameter) from 7-day-old fungal pathogen cultures were placed at the centre of the PDA base plate. Antagonist and pathogen plates were sealed together with Parafilm and incubated at 25 °C. Control plates devoid of *A. pullulans*, were treated with SDW (100 µL). The diameter of the pathogen colony was measured after three days of incubation at four radial distances and averaged. Six replicates were performed for each antagonist-pathogen combination and controls.

#### Inhibition of conidia germination

A conidial suspension of either *B. cinerea* or *A. alternata* (10^6^ spores/mL, 30 µL), in potato dextrose broth (PDB), was placed on a glass microscope slide, in a Petri dish lined with a moistened filter paper. A second Petri dish containing a 48 h culture of *A. pullulans* growing on PDA was placed face-down on the base plate containing the microscope slide with the fungal pathogen. Control plates were prepared as described above. Plates were sealed and incubated for 5 h at 25 °C in darkness. Two hundred conidia were assessed for germination and the results expressed as a percentage. Conidia were considered as germinated when the germ tube length was equal to or greater than the transverse diameter of conidia and there was no observable disruption. Three replicates were performed.

### Analysis of *A. pullulans* VOCs by headspace SPME-GC-MS

#### Experimental set up for SPME automated sampling of VOCs

**Figure 1:**
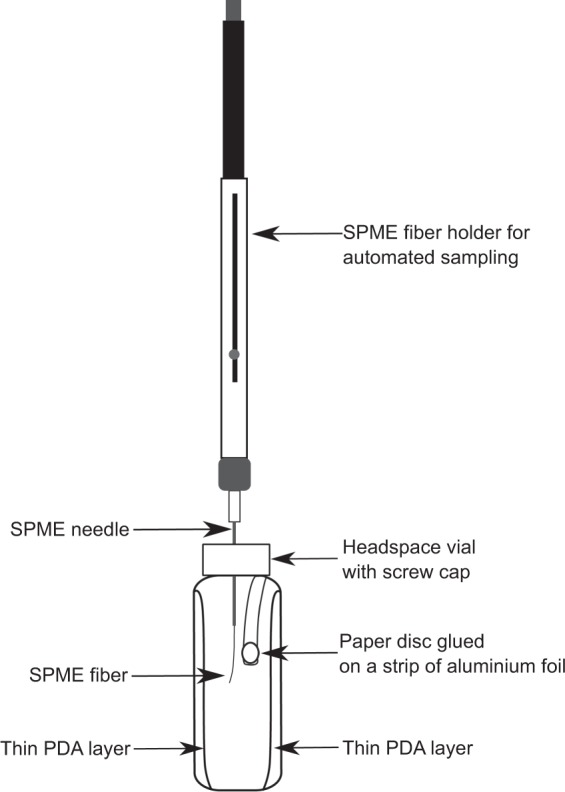
Culture headspace sampling setup to directly extract and quantify VOCs from an antagonist and pathogen system by automated SPME-GC-MS.

PDA (1.5 mL) was poured into a 20 mL headspace vial laying on its side such that the agar solidified on the vertical wall of the vial. The vial was then rotated and a further volume of PDA (1.5 mL) was poured on to the opposite side of the vial, in the same way the next day ensuring that the two layers were not mixed. A cell suspension of *A. pullulans* in SDW (10^8^ cells/mL, 10 µL) was spread on to one of the PDA layers of the 20 mL headspace vial. The vial was sealed with a screw cap with PTFE septum and incubated in a horizontal position at 25 °C for 48 h. The opposite PDA layer was then inoculated with a spore suspension of either *B. cinerea* or *A. alternata* (10^6^ spores/mL, 10 µL). A paper disc (10 mm, Sigma-Aldrich, MO, USA) glued (PVA based glue) on a strip of aluminium foil and heated at 120 °C in an oven for 2 h to volatilise any compounds present in the adhesive, was hung on the wall of the headspace vial. The disc was used to add the IS immediately before the GC-MS analysis (Fig. 1). Care was taken to keep the strip away from the PDA layer and the length was kept to approximately 1 cm in order to prevent contamination of the SPME fibre. After pathogen inoculation, the vials were incubated for 72 h at 25 °C. Each pathogen-antagonist combination consisted of four types of growth each with four replicates; negative antagonist (*A. pullulans* only), negative pathogen (either *B. cinerea* or *A. alternata* only) interaction (inoculated with both *A. pullulans* and respective pathogen), and the blank (non-inoculated media). The following antagonist-pathogen combinations were grown in interaction vials, *A. pullulans* A1-*B. cinerea* TN080, *A. pullulans* A2-*B. cinerea* DAR69764, *A. pullulans* A2-*A. alternata* and *A. pullulans* A3 with either *B. cinerea* TN080, *B. cinerea* DAR69764 or *A. alternata*.

An IS comprising 2-methyl-4-pentanol in methanol (40 mg/L, 5 µL) was added to the disc, using a glass syringe (Hamilton syringe^**®**^) with Chaney adapter through the septum of the headspace vial cap, immediately before the GC-MS analysis. The concentration, volume and type of IS were decided and adjusted from a series of preliminary injections (data not shown), in order to produce a peak height in the chromatogram at approximately the midpoint of peaks height for the unknown VOCs.

Headspace vials containing samples were placed in a Peltier cooled sample tray at 10 °C which minimised further growth of organisms and reduced the impact on changing headspace composition, and were sampled in a random order. Vials were transferred into a heated (40 °C) block and incubated for 1 min. A 2 cm SPME fibre with 50/30 µm DVB/CAR/PDMS (Supelco, PA, USA) coating was inserted into the headspace of the sample vial and exposed to the headspace for 15 min at 40 °C.

#### GC-MS analysis

VOCs analysis was performed with a 7890B gas chromatography system (Agilent, Palo Alto, CA, USA) equipped with a CombiPal auto-sampler (CTC Analytics, Zwingen, Switzerland) coupled to an Agilent 5975C triple quadrapole mass detector. The SPME fibre was desorbed into an ultra-inert straight SPME liner (0.75 mm, Agilent Technologies Inc., USA) at 250 °C in splitless mode for 2 min, and separation of compounds achieved through a DB-Waxetr column (60 m × 250 µm inner diameter × 0.25 µm film thickness; Agilent Technologies Ltd., USA) with helium at a flow rate of 1 mL min^−1^. The oven temperature was set to 40 °C for 3 min and then ramped from 40 to 90 °C at 10 °C min^−1^, 90 to 180 °C at 5 °C min^−1^, 180 to 250 °C at 20 °C min^−1^ and held for 2 min, resulting in a total run time of 31.5 min. The mass spectrometer was operated in an electron impact (EI) ionization at 70 eV with an ion source temperature of 250 °C, to scan a mass range from 35 m/z to 350 m/z.

#### Data processing

The total ion chromatogram (TIC) of all samples were overlaid in Agilent MassHunter Qualitative analysis B.07.00 software to identify appropriate time windows for automated processing. Each time window was selected along a stable base line including two to three peaks with a profile height not less than 10^6^ abundance. The GC-MS data files (.d) were converted into excel (.xlsx) files with Openchrom (community edition 1.2.0.) and imported into Matlab (version R.2018b, The Mathworks Inc., MI, USA). Files were processed according to previously published MCR-ALS analysis procedures^23,27^.

Siloxane related mass ions, including 73, 133, 147, 149, 191, 193, 207, 208, 209, 221, 249, 265, 267, 268, 269, 281, 282, 283, 295, 325, 327, 341, 342^27^ were excluded from the data matrix as they were due to SPME fibre breakdown products. Spectra extracted during the chromatogram deconvolution by MCR-ALS were exported from Matlab in a format compatible with the National Institute of Standards and Technology (NIST) Mass Spectral Search Program, 2014, version 2.2 (USA), and a peak table with areas associated with extracted variables including the IS, used for subsequent multivariate analysis.

#### Identification of compounds

The spectra imported in to NIST−14 library and variables were tentatively identified by comparing with those reference compounds from the library. Any known artificial peaks were excluded from the data set. The compounds with more than 80% of similarity with reference spectra were selected. The production of these selected compounds by *A. pullulans* were confirmed using box-plots (Supplementary material Fig. S5) of feature (VOCs) abundances after normalisation to the IS from MCR-ALS extracted data. The identity of those selected compounds were confirmed by comparison with the retention time of authentic standards run in identical GC-MS conditions. Linear retention indices (LRI) were calculated using *n*-alkane series (C8−C20) run in similar oven ramp and gas flow conditions and the equation of van den Dool and Kratz and compared with literature^28^.

#### Sample classification by PCA and PLS-DA

An offset of 1 was added to all m/z channels of the normalised data matrix of variables followed by log 10 transformation, mean-centering and Pareto-scaling prior to principal component analysis (PCA). Scores were plotted with samples coded for negative antagonist, negative pathogen, interaction and blank.

Subsequently, data from *A. pullulans* A1-*B. cinerea* TN080, *A. pullulans* A2-*B. cinerea* DAR69764 and *A. pullulans* A2-*A. alternata* (assigned to class interaction) and combined data from negative pathogen and blanks (assigned to class blank) were subjected to a partial least squares-discriminant analysis (PLS-DA) as a supervised classification method. PLS-DA was conducted using two latent variables. PLS-DA model efficacy was evaluated using a twofold evaluation. Firstly, model cross-validation^29^ was performed using a subset resampling methodology by omitting random subsets of the samples with ten data splits. Cross-validation was followed with a Monte Carlo permutation of the data class with the null hypothesis being no predictive acuity. Metrics of true model performance (Q^2^, area under the receiver operator curve (AUROC) and number of misclassifications) were compared to the permuted models (1,000 iterations) and empirical p-values determined from the proportion of test statistics from the random models equal to or higher than the true model^30^. Results of the permutation testing are presented in the supplementary material (Supplementary material Figs. S1,S2).

Selectivity ratios (SR)^31^ and variable importance in projection (VIP)^31^ scores from the PLS-DA model were used to determine the most important variables responsible for the discrimination between sample groups. Peaks with a VIP score of greater than one and with high SR scores were considered important target compounds for quantification and interaction studies.

### Quantification of *A. pullulans* VOCs

Calibration curves (R^2^ > 0.99) were prepared for ethanol, 2-methyl-1-propanol, 3-methyl-1-butanol and 2-phenylethanol. A vial of target compounds in a water matrix (5 mL) was spiked with IS (2-methyl-4-pentanol, 0.04 mg/L) and analysed using SPME-GC-MS conditions as described above. At least six concentrations of each compound were used with two replicates. Concentrations of the four analytes in the interaction sample headspace were calculated.

### Antifungal activity and synergistic effects of VOCs

The concentrations of compounds needed to be added to the mixture used for RSM were determined using a series of preliminary experiments as the PDA potentially absorb compounds which would confound the analytical results reliant on headspace concentration. Each compound mixture (500 µL) prepared in water, was added to a filter paper (Whatmann No. 1, 85 mm diameter) placed on one side of a Petri dish and covered immediately with a base Petri dish containing PDA (10 mL). The two dishes were immediately sealed with Parafilm and incubated at 25 °C. After three days of incubation, the headspace was manually sampled by SPME-GC-MS. The IS (2-methyl-4-pentanol, 330 mg/L, 5 µL) was spotted onto a paper disc glued on aluminium foil as described above and inserted into the double Petri dish setup immediately before sampling through a small pre-cut open of the Parafilm, keeping any loss of compounds to a minimum. The SPME fiber was manually inserted into the double Petri dish setup through a previously prepared hole on a side of the Petri dish and kept for 15 mins. The fibre was retracted, desorbed into the GC/MS inlet and VOCs were analysed using the same oven ramp conditions described above. The normalised peak areas of the four compounds in the double Petri dish headspace were compared with the normalised peak areas obtained from the original *A. pullulans* culture headspace in vials as the IS concentration in the two headspaces were identical. When the two normalised peak areas share a similar range, those concentrations added to double Petri dish setups were selected for the following RSM experiment.

#### Response surface modelling (RSM)

A Box–Behnken experimental design was applied to investigate the synergistic inhibitory effects of the four compounds (ethanol, 3-methyl-1-butanol, 2-methyl-1-propanol and 2-phenylethanol) on the growth of *B. cinerea* and *A. alternata*. A total of twenty seven experiments were performed, including three centre points. All variables were in three-coded factor levels: −1, 0, +1, corresponding to the low, mid and high levels respectively (Supplementary material Table S1). The top PDA plate of the double Petri dish was inoculated with a pathogen mycelial plug. The compound mixture (500 µL) was added to a filter paper in the bottom Petri dish. Colony diameter was measured after three days of incubation at 25 °C.

The following quadratic polynomial equation was fitted to model the relationship between four variables and the response,

1 $$\begin{array}{rcl}{\rm{Y}} & = & {{\rm{\beta }}}_{0}+{{\rm{\beta }}}_{1}{\rm{A}}+{{\rm{\beta }}}_{2}{\rm{B}}+{{\rm{\beta }}}_{3}{\rm{C}}+{{\rm{\beta }}}_{4}{\rm{D}}+{{\rm{\beta }}}_{12}{\rm{AB}}+{{\rm{\beta }}}_{13}{\rm{AC}}+{{\rm{\beta }}}_{14}{\rm{AD}}+{{\rm{\beta }}}_{23}{\rm{BC}}\\  &  & +\,{{\rm{\beta }}}_{24}{\rm{BD}}+{{\rm{\beta }}}_{34}{\rm{CD}}+{{\rm{\beta }}}_{11}{{\rm{A}}}^{2}+{{\rm{\beta }}}_{22}{{\rm{B}}}^{2+}{{\rm{\beta }}}_{33}{{\rm{C}}}^{2}+{{\rm{\beta }}}_{44}{{\rm{D}}}^{2}\end{array}$$

where A, B, C and D represent the coded values of ethanol, 3-methyl-1-butanol, 2-methyl-1-propanol and 2-phenylethanol respectively. Y is the predicted response, β_0_ is the intercept term, β_i_ is the linear coefficient, β_ij_ is the interaction coefficient and β_ii_ is the quadratic coefficient. Depending on the significant terms and other statistical parameters of validation, models were simplified and fitted by suppressing quadratic terms and certain interaction terms which were not statistically significant (P > 0.05). Simplified final models are presented in the results section. Design generation, analysis and optimisation were carried out using Design Expert STAT Ease software 7.0 version (Minneapolis, USA).

### Statistical analysis

The data from the mycelium growth inhibition assay and the conidia germination assay were subjected to an analysis of variance (ANOVA) with Tukey’s mean separation test to evaluate significant differences among treatments at a 95% confidence level. Analyses were conducted using SPSS ver. 24.0 (IBM SPSS Statistics 24).

## Results

### Reduced mycelium growth and conidia germination of *B. cinerea* and *A. alternata* by *A. pullulans* VOCs

#### Mycelium growth

**Figure 2:**
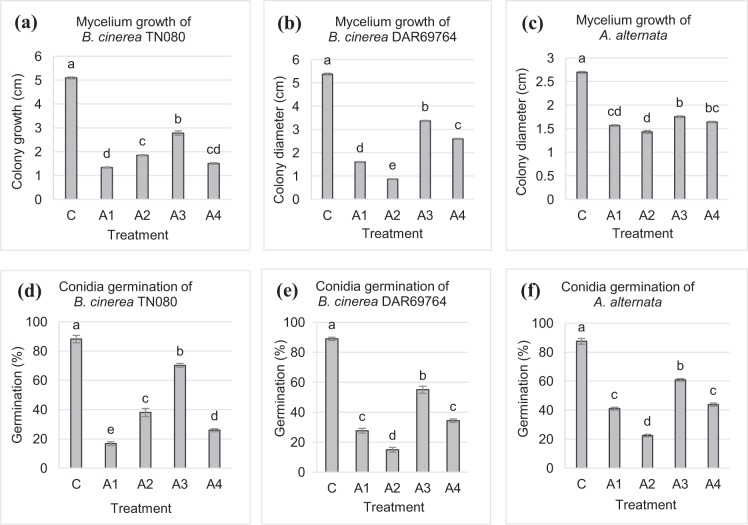
The effect of VOCs produced by isolates of *A. pullulans* (A1–A4) on the mycelium growth and conidia germination. Mycelium growth of (**a**) *B. cinerea* TN080, (**b**) *B. cinerea* DAR69764 and (**c**) *A. alternata* after three days of incubation. Error bars indicate standard error of mean of the six replicates. Suppression of conidia germination of (**d**) *B. cinerea* TN080, (**e**) *B. cinerea* DAR69764 and (**f**) *A. alternata* by exposure to VOCs of *A. pullulans*. Error bars indicate standard error of mean of the three replicates. In every graph, ‘C’ represents the control. Data of the mycelium growth and conidia germination were subjected to analysis of variance. Different letters represent significant differences (P < 0.05) explained by one way ANOVA with Tukey’s mean separation test.

VOCs of the four *A. pullulans* isolates significantly inhibited (P < 0.05) the colony growth of the three pathogens. The degree of inhibition after three days of incubation varied between isolates (Fig. 2a–c). *A. pullulans* A1 VOCs resulted in the highest inhibition of *B. cinerea* TN080, whereas *B. cinerea* DAR69764 and *A. alternata* were highly inhibited by *A. pullulans* A2 VOCs. In comparison with controls, these inhibitions were 74%, 84% and 47% for *B. cinerea* TN080, *B. cinerea* DAR69764 and *A. alternata* respectively. Among the four *A. pullulans* isolates examined in this study, *A. pullulans* A3 headspace inhibited the growth of *B. cinerea* TN080, *B. cinerea* DAR69764 and *A. alternata* the least,(Fig. 2a–c).

#### Conidia germination and morphological deformations

**Figure 3:**
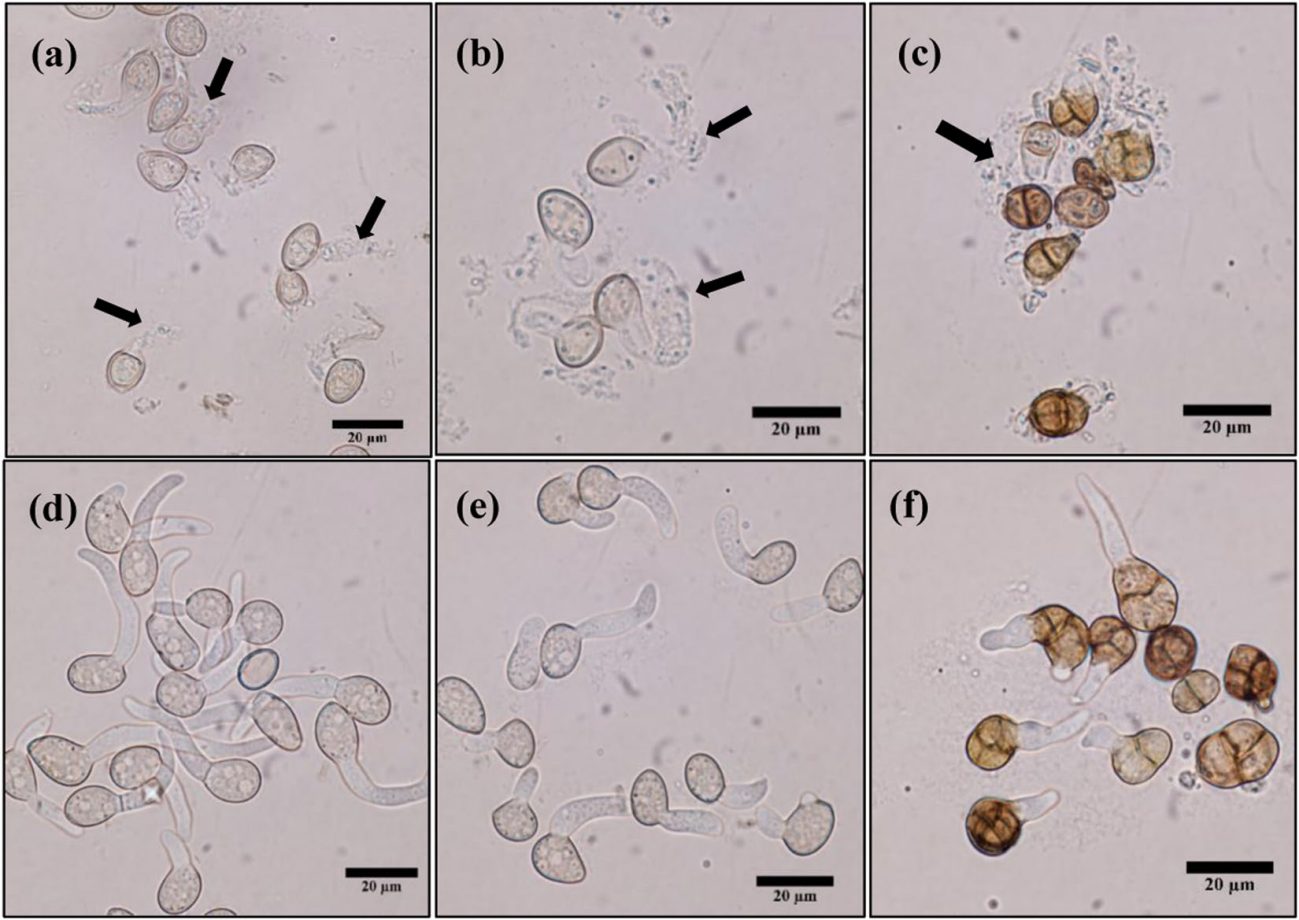
Deformed germ tubes and conidia upon exposure to *A. pullulans* VOCs; (**a**) *B. cinerea* TN080 with *A. pullulans* A1 VOCs, (**b**) *B. cinerea* DAR69764 with *A. pullulans* A2 VOCs and (**c**) *A. alternata* with *A. pullulans* A2 VOCs, in comparison with non-fumigated controls; (**d**) *B. cinerea* TN080, (**e**) *B. cinerea* DAR69764 and (**f**) *A. alternata*. Arrow heads indicate disrupted germ tubes and conidia.

VOCs of the four *A. pullulans* isolates supressed conidia germination of the three pathogens to various degrees (Fig. 2d–f). These results were consistent with the mycelium growth inhibition assay. Conidia germination of *B. cinerea* TN080 was highly inhibited by *A. pullulans* A1 headspace whereas the highest inhibition of conidia germination of *B. cinerea* DAR69764 and *A. alternata* were found when exposed to *A. pullulans* A2 VOCs. The least inhibition of conidia germination was observed in the presence of *A. pullulans* A3 against all three pathogen isolates. Light microscopic images of pathogen conidia exposed to headspace of the highest inhibitory *A. pullulans* isolates; *B. cinerea* TN080 with *A. pullulans* A1 VOCs (Fig. 3a), *B. cinerea* DAR69764 with *A. pullulans* A2 VOCs (Fig. 3b), and *A. alternata* with *A. pullulans* A2 VOCs (Fig. 3c), revealed that exposure to *A. pullulans* VOCs may cause damage to the cell wall and cell membrane, resulting in the release of cellular content.

### PCA discriminated between *A. pullulans* inoculated and non-inoculated headspace

Based on the results of the two antifungal assays described above, *A. pullulans* isolates were selected for VOCs analysis, *A. pullulans* isolate A1 which inhibited *B. cinerea* TN080, isolate A2 which inhibited *B. cinerea* DAR69764 and *A. alternata* the greatest and *A. pullulans* isolate A3 which had the least inhibitory activity towards all fungal pathogens examined in this study.

**Figure 4:**
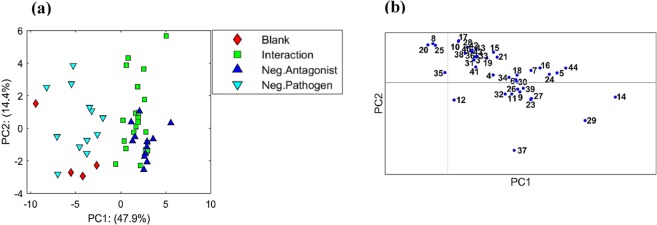
(**a**) PCA scores plot of GC-MS normalised peaks obtained from culture vial headspace of 52 samples, showing PC1 vs. PC2. Four categories of samples on PCA scores plot include blank (non-inoculated media), interaction (inoculated with both *A. pullulans* and respective pathogen), negative antagonist (either *A. pullulans* A1 or *A. pullulans* A2 or *A. pullulans* A3 only) and negative pathogen (either *B. cinerea* TN080 or *B. cinerea* DAR 69764 or *A. alternata* only) each with four replicates. (**b**) PCA loadings plot shows PC1 vs. PC2 with forty one variables identified from culture headspace. Variables include volatile substances produced by the antagonist, pathogen, culture medium and potential fiber and column contaminants.

MCR-ALS analysis resulted in peak areas of forty three compounds from culture headspace comprising volatile substances produced by the antagonist (three *A. pullulans* isolates), pathogen (*B. cinerea* TN080, *B. cinerea* DAR 69764 and *A. alternata*), culture medium and potential fibre and column contaminants. The normalised peak table was used for PCA. The first latent variable of the scores plot shows the separation along PC1 (Fig. 4a), describing blank and negative pathogen samples separation from negative antagonist and interaction samples. This explains 48% of data variance along PC1. As PCA is an unsupervised method, the clear separation between the two groups instinctively confirms differences in the volatile composition of headspace between antagonist inoculated and non-inoculated samples. The corresponding loadings plot (Fig. 4b) shows variables located on the right side from the origin in PC1 influence the discrimination of *A. pullulans* inoculated samples where compounds 14, 29 (Table 1) and 37 seem to have a greater influence on this discrimination. There was no clear discrimination between negative antagonist and interaction samples (Fig. 4a).

**Table 1:** Volatile organic compounds from *A. pullulans* culture headspace identified using authentic standards (Std), comparison with mass spectra from NIST (MS) and comparison with LRI from peer reviewed publications.

Peak No. in PCA loadings plot	VOC	LRI_(calculated)_	LRI_(Lit)_	Identification level
5	Ethanol	928	928^55^	MS, Std, LRI
16	2-Methyl-1-propanol	1,092	1,092^56^	MS, Std, LRI
24	3-Methyl-1-butanol	1,162	1,208^55^	MS, Std, LRI
26	2-Heptanol	1,250	1,312^57^	MS, Std, LRI
34	2-Nonanol	1,461	1,511^57^	MS, Std, LRI
44	2-Phenylethanol	1,841	1,880^58^	MS, Std, LRI
29	3-Methyl-1-hexanol	1380	1423^59^	MS, LRI
3	Acetone	—	820^60^	MS, Std
23	2-Heptanone	1,152	1,168^61^	MS, Std, LRI
27	2-Nonanone	1,356	1,373^61^	MS, Std, LRI
6	Ethyl acetate	—	905^56^	MS, Std
14	Ethyl butyrate	1,037	1,029^62^	MS, Std, LRI
18	3-Methylbutyl acetate	1,114	1,117^56^	MS, Std, LRI
11	2-Methylpropyl acetate	1,014	1,003^62^	MS, Std, LRI
9	Unknown	—	—	MS

### Identification of fourteen VOCs from *A. pullulans* headspace

MCR-ALS extracted spectra for forty four variables (including IS) were imported to NIST mass spectral library and were tentatively identified by comparing with those reference spectra from the library. Fifteen compounds were filtered out with greater than 80% of similarity to those of reference compounds and appear to be produced by *A. pullulans* (Table 1). Seven alcohols, three ketones and four esters were among the fifteen compounds. One compound was reported as unknown (Table 1). The identity of these compounds were further confirmed by comparing their retention times with authentic standards and with LRI derived from the alkane series run in an identical GC oven ramp. These fifteen compounds were produced by all three isolates of *A. pullulans* (Supplementary material Fig. S5).

### PLS-DA with VIP and SR tests identified four important VOCs for sample discrimination

PLS-DA was performed using peak areas of two sample classes (interaction and blank). According to the VIP and SR scores, four compounds possessed the greatest explanatory power when separating the *A. pullulans* inoculated interaction samples from blanks along PC1. These compounds were ethanol, 2-methyl-1-propanol, 3-methyl-1-butanol and 2-phenylethanol.

The quality of the fitted model was excellent as shown in Supplementary material Figs. S1,S2. There is a clear distinction between the permuted distribution and the original data based on number of misclassifications, the Q^2^ values and the AUROC values for both classes (interaction and blank). The AUROC values close to 1 indicate a perfect separation between the classes. The significant p values obtained for Q^2^ values (P = 0.001 for class interaction, P = 0.024 for class blank) and number of misclassifications (P = 0.001 for class interaction, P = 0.024 for class blank) based on the differences in the permuted and true data, indicate a good predictability of the applied model.

### Quantified *A. pullulans* VOCs in the interaction headspace

Four VOCs identified from the PLS-DA with high discriminatory value were quantified in the interaction headspace (Table 2). As observed in Table 2, ethanol was produced in extremely high concentrations in all three systems when compared to the other three VOCs.

**Table 2:** Concentration (mg/L) of *A. pullulans* VOCs in antagonist-pathogen interaction culture headspace.

Compound	*A. pullulans* (A1) + *B. cinerea* TN080	*A. pullulans* (A2) + *B. cinerea* DAR69764	*A. pullulans* (A2) + *A. alternata*
Ethanol	397	459	524
2-Methyl-1-propanol	1.2	1.3	2.3
3-Methyl-1-butanol	1.0	1.2	1.9
2-Phenylethanol	2.5	2.2	3.6

### RSM optimised four antifungal VOCs to synergistically minimise *B. cinerea* and *A. alternata* colony growth

**Figure 5:**
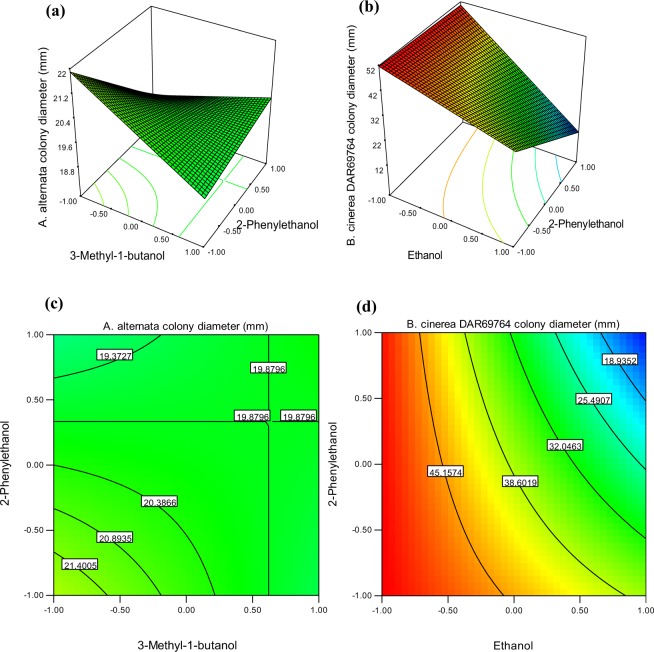
Response surface and contour plots showing significant interactive pairs of VOCs. (**a**) Response surface between 3-methyl-1-butanol and 2-phenylethanol, (**c**) contour plot between 3-methyl-1-butanol and 2-phenylethanol, show a significant interaction against *A. alternata* colony growth. (**b**) Response surface between ethanol and 2-phenylethanol and (**d**) contour plot between ethanol and 2-phenylethanol show a significant interaction on *B. cinerea* DAR69764 colony growth reduction. Colony diameter reduces from red to blue on response surfaces and contour plots. Plots have created using the coded levels of concentrations for each VOC.

Concentrations of ethanol, 2-methyl-1-propanol, 3-methyl-1-butanol and 2-phenylethanol were optimised to minimise colony growth of *A. alternata* and *B. cinerea* using a Box-Behnken experimental design followed by RSM (Fig. 5, Table 3). The following simplified polynomial response surfaces were fitted on colony diameter of (Y);

2 $${\rm{Y}}(A.alternata)=20.07-4.46{\rm{A}}-0.31{\rm{B}}+0.19{\rm{C}}-0.587{\rm{D}}+0.94{\rm{BD}}$$

3 $${\rm{Y}}\,(B.cinerea\,{\rm{TN}}080)=34.73-12.25{\rm{A}}-1.92{\rm{B}}-1.63{\rm{C}}-2.63{\rm{D}}$$

4 $${\rm{Y}}\,(B.cinerea\,{\rm{DAR}}69764)=38.05-13.10{\rm{A}}-1.06{\rm{B}}-1.77{\rm{C}}-6.56{\rm{D}}-6.00{\rm{AD}}$$

**Table 3:** Optimised concentrations (mg/L) of VOCs to reduce colony growth of *B. cinerea* and *A. alternata* determined by RSM.

VOC	Optimised concentration by RSM (mg/L)	
	*A. alternata*	*B. cinerea* TN080 and *B. cinerea* DAR69764
Ethanol	322,500	200,000
3-Methyl-1-butanol	850	2,975
2-Methyl-1-propanol	1,700	3,238
2-Phenylethanol	3,250	3,213

Model F-values 81.51 (Eq. (2)), 92.07 (Eq. (3)) and 21.08 (Eq. (4)) were significant using p ≤ 0.05. Lack of fit F-values were non-significant (P > 0.05) and residuals were normally distributed for the three models. R^2^ values 0.9575 (Eq. (2)), 0.9456 (Eq. (3)) and 0.8001 (Eq. (4)) obtained for the three models, indicated that these models were accurate for predicting responses. The main effects of the compounds and second order interactions were determined based on the fitted equations. The sign of the β coefficients of the polynomial surface response indicated a positive (increased growth) or negative (reduced growth) impact on the colony growth. The size of the effect was determined from the level of significance expressed by the p-value (Supplementary material Table S2).

The colony diameter of *A. alternata* was significantly reduced by ethanol (P < 0.0001, term A) and 2-phenylethanol (P = 0.0170, term D). However, the interaction between these two compounds was not significant (P > 0.05), thus this term was removed from the final model (Eq. (2)). The interaction between 3-methyl-1-butanol and 2-phenylethanol caused a significant (P = 0.0255) positive impact which is an increased growth. The response surface plot (Fig. 5a) and contour plot (Fig. 5c) of 3-methyl-1-butanol and 2-phenylethanol indicated a reduction of the diameter of *A. alternata* when one of the two compounds of the pair is at its highest concentration and the other compound is at the lowest concentration. According to the fitted linear model (Eq. (3), Supplementary material Table S2), all four compounds individually exert a significant effect (P < 0.05) on the colony growth reduction of *B. cinerea* TN080. None of the second order interactions between these compounds were significant in terms of the impact on colony growth. *B. cinerea* DAR69764 colony growth was significantly reduced by ethanol (P < 0.0001, term A) and 2-phenylethanol (P = 0.0002, term D) (Supplementary material Table S2). The interaction between the two compounds was also significant (P = 0.0293). According to the response surface plot (Fig. 5b) and contour plot (Fig. 5d) the lowest colony diameter was observed when both compounds were at their highest concentrations. The three linear models were validated by testing mixtures of three different concentrations of the four compounds.

The minimum colony diameters predicted by RSM were 14.5 mm, 16.84 mm and 10.14 mm for *A. alternata*, *B. cinerea* TN080 and *B. cinerea* DAR69764 respectively. The concentrations of the four compounds were optimised together for the two *B. cinerea* isolates. The optimised concentrations of the four compounds in the mixture can be observed in Table 3.

## Discussion

Fungicides and other chemical-based methods for plant disease management have become less popular in recent years due to public concern about environmental pollution, issues with pesticide resistance and the removal of pesticide registrations^32^. The utilisation of biocontrol agents such as microbial VOCs represent a more environmentally friendly^4,5^ alternative for plant disease management. Research on microbial VOCs fumigation has demonstrated successful antifungal effects on a large spectrum of pathogenic fungi and bacteria^3,33^. Despite these advances, plant diseases caused by *B. cinerea* and *A. alternata* remain a challenge^14,34^.

*A. pullulans* has been widely studied as a biological control agent^7,8^. A variety of mechanisms have been postulated to describe its antifungal properties including enhanced natural host defences^7^, competition for nutrients^9,35^ and antifungal VOCs production^11^. The latter has been a recently investigated area and not well characterised to date. In order to better describe the biological control potential of *A. pullulans* against *B. cinerea* and *A. alternata*, its ability to produce antifungal VOCs was studied by incorporating a chemometrics approach.

Knowing the negative effects of microbial VOCs on fungal physiological functions including mycelial growth, spore germination, sporulation and sclerotial development^11,33^, we studied the antifungal effects of *A. pullulans* VOCs on two fungi *B. cinerea* and *A. alternata*, responsible for disease losses in a wide range of agricultural crop plants. We observed a strong reduction of pathogen mycelial growth and conidia germination of the two pathogens by *A. pullulans* VOCs. A notable, less dense and weak mycelial mat was evident and a clear disruption of conidial germ tubes was noted in exposed mycelia and conidia (Fig. 3a–c).

Previous studies on volatile analysis have identified a number of VOCs in the *A. pullulans* culture headspace^12^. In our study, headspace-SPME-GC-MS analysis incorporated with MCR-ALS revealed fifteen VOCs produced by *A. pullulans* and fourteen of them were identified. Thus, the observed inhibitory activity is due to the activity of a set of *A. pullulans* VOCs which include alcohols, ketones and esters. 3-Methyl-1-butanol, 2-methyl-1-propanol and 2-phenylethanol, identified from *A. pullulans* headspace in our study were previously recorded from *A. pullulans* headspace and were also tested against *B. cinerea*, *Penicillium* and *Colletotrichum* sp. and observed notable inhibitory activities^11^. To the best of the authors’ knowledge, 3-methyl-1-hexanol, acetone, 2-heptanone, ethyl butyrate, 3-methylbutyl acetate and 2-methylpropyl acetate were identified for the first time to be produced by *A. pullulans* isolates. However, these compounds have been previously reported in the culture headspace of other microbial organisms: 3-methyl-1-hexanol from *Lysobacter* species^36^, 2-heptanone from *Escherichia coli*^37^, 3-methylbutyl acetate from *Sporobolomyces roseus*^12^, acetone from *Muscodor albus*^24^ and ethyl butyrate and 2-methylpropyl acetate from *Saccharomyces cerevisiae*^38^.

Quantitation of VOCs is as crucial as identification, especially in the evaluation of antifungal activity of VOCs in artificial cocktails. Quantification studies of microbial VOCs so far has mainly been based on relative peak areas of compounds on chromatograms^17,39^ while some of the studies tested random mixtures with various concentrations^40^. However, a lack of IS makes a comparison of VOCs efficacy challenging mainly due to variations in the SPME performance between injections^19^. As SPME technique, does not reflect the real situation, previous studies have also mentioned the importance of using standards to quantify real VOCs atmosphere concentration^11^. We observed an area ratio of 2:1:1.2 between ethanol: 3-methyl-1-butanol and 2-phenylethanol while actual quantification using authentic standards and IS resulted in a ratio of 397:1:2.5 for the three compounds respectively in the *A. pullulans* (A1) and *B. cinerea* TN080 interaction headspace. This indicates the necessity of accurate quantification of VOCs. A previous study reported that *Muscodor albus* VOCs did not mimic the natural atmospheric conditions on some of the test organisms such as *Candida albicans*, *Sclerotinia sclerotiorum* and *Aspergillus fumigatus*^24^. The authors suspected a key ingredient was missing in the artificial test atmosphere to cause a biocidal activity on those fungal cells. However, artificial volatile formulations based on incorrect proportions could be an alternative possibility for dissimulation of the natural atmospheric conditions.

Our study introduces a novel setup (Fig. 1) to directly extract VOCs from the culture headspace by automated SPME-GC-MS analysis, where both antagonist and pathogen are present simultaneously and actively interacting; unlike previous studies, we also used an IS to facilitate the quantification of VOCs. This is the first report to introduce a direct system for automated extraction of microbial VOCs from a dual culture setup which also facilitates quantification of volatiles. In a previous study^41^, microbial VOCs quantification was carried out using IS, in which aliquots of the original liquid cultures were transferred to headspace vials and an IS was added followed by SPME-GC-MS analysis. Headspace analysis of the original culture provides a reliable quantitative approach, as it prevents losing a portion of VOCs during transfer, which was actually produced in the culture and partitions into the headspace. This is important, particularly given that volatility varies among different compounds. In this regard, the technique proposed here represents a more reliable and robust approach for the accurate quantification of microbial VOCs. As we observed in the preliminary experiments of the present study, selecting suitable IS which are non-inhibitory to the test organisms and persist in the culture system throughout the growth period without being absorbed by the mycelia is challenging. These reasons may have discouraged the application of IS in previous bio-control studies. Moreover, a promising method to introduce a suitable IS to a culture system without disturbing its headspace upon completion of the growth period is required. Our study has introduced a reliable, robust approach to overcome this issue (Fig. 1).

When combined the untargeted feature extraction process with PLS-DA it is possible to determine which features, or combination of features, from the extracted data table of peak areas enable separation of specific sample groups when these are modelled. By numerically defining specific class membership (−1 or +1) and using a projection to latent structures approach, the PLS algorithm seeks to predict the class membership by maximising the co-variance between the peak area table and the class membership values. In the present investigation we defined class membership for PLS-DA as ‘media blank’ + ‘pathogen’ and ‘interaction samples’ being those samples with growth of the antagonist and pathogen. Effectively peak areas that are derived from the media blank and from growth of the pathogen only, are mathematically subtracted from the peak areas of the remaining samples so that discrimination of the modelled classes is achieved. Thus, PLS-DA successfully discriminated between *A. pullulans* inoculated interaction samples and the non-inoculated culture headspace (Fig. 4b). Permutation testing metrices of Q^2^, AUROC and number of misclassifications (Supplementary material Fig. S1,S2) confirmed the robustness of the true model to explain the discrimination between *A. pullulans* and pathogen inoculated class interaction and the class blank (P < 0.001). Ethanol, 2-methyl-1-propanol, 3-methyl-1-butanol and 2-phenylethanol were identified as the most effective VOCs to discriminate two sample classes (Fig. 4b). Our RSM (Fig. 5, Supplementary material Table S2) with these four VOCs revealed, ethanol and 2-phenylethanol have significant effects on supressing colony growth of both *B. cinerea* and *A. alternata*. Significant differences are evident between the different antagonist isolates and controls for inhibition of the pathogen mycelial growth. This is in agreement with the PCA scores plot (Fig. 4a) which shows a clear discrimination of blanks and pathogen samples from *A. pullulans* and interaction samples along PC1 axis. No clear separation between different *A. pullulans* isolates or between specific antagonist-pathogen combinations is apparent (Supplementary material Fig. S3).

The efficacy of ethanol as a biocontrol agent has been previously demonstrated in other studies. Previous work on ethanol fumigation on table grapes at low temperature postharvest storage successfully controlled *B. cinerea*^42,43^. Also ethanol in combinations with other alternatives such as potassium sorbate^44^ and calcium chloride^42^, was used to improve disease control against *B. cinerea*. Exposure to ethanol solutions at low temperatures reduced spore germination of *A. alternata*^43^. However, 1,000 µg/mL of ethanol only had a transitory inhibitory effect on *A. alternata*^45^ which agrees with our findings that the optimised concentration of ethanol by RSM is in the order of 10^6^ to control *A. alternata in vitro*. This means ethanol could be effective in extremely high concentrations. Likewise, 2-phenylethanol has also been demonstrated to have antimicrobial activity against various pathogenic fungi such as *Penicillium italicum*^46^, *Aspergillus flavus*^47^, *B. cinerea* and *Colletotrichum acutatum*^11^.

Various mechanisms have been hypothesised for microbial VOCs mediated antifungal effects. 2-Phenylethanol mediated inhibition of *P. italicum* cells identified to be highly associated with interactions with the mitochondria and the nucleus^47^. The mechanism behind the toxicity of alcohols, including ethanol and larger alkanols is likely due to their role in membrane disruption, leading to the dissipation of the proton gradient of affected cells^48^ which subsequently interferes and/or inhibits regular cell metabolic functions^49^.

There are differences in sensitivity to these compounds among fungal species^50,51^. Thus, optimal treatments for different fungal pathogens will be different. Differential sensitivity could be explained by differences in the composition of membranes (e.g. lipid to protein ratio) and cell walls between fungal species and involvement of other consequences such as oxidative stress and imbalanced cellular redox homeostasis to different degrees upon volatile fumigation^52^ and impacts on enzymes related to energy-generating pathways and metabolism^53^. This may explain why we found *A. alternata* was less sensitive to *A. pullulans* VOCs than *B. cinerea*.

The use of RSM in our study revealed interactive effects between *A. pullulans* VOCs in the inhibition of *B. cinerea* and *A. alternata* colony growth. Many studies report a single VOCs is not lethal to a particular pathogen when studied in isolation, and hypothesise a synergistic or additive effect of different compounds^24^. Microbial VOCs are produced at low concentrations by organisms, which also suggest their effect to be synergistic or additive^25^. These assumptions were further confirmed in our study (Fig. 5). Interestingly, 3-methyl-1-butanol^11,54^ and 2-phenylethanol^11,46^ which are two well-known antifungal compounds, increased the growth of *A. alternata* when present in an interaction whereas their individual effects were negative. Further experiments are essential to confirm and investigate the mechanisms behind these positive interactive effects.

There are instances where artificial environments did not fully replicate the inhibitory effects of pathogens which were observed in natural antagonist headspace^16,24^. In addition to relying on relative peak areas of VOCs, the absorption of VOCs to the culture medium also needs to be considered when preparing accurate formulations of artificial VOCs mixtures for antifungal assays. However, no prior studies appear to have considered this effect. According to our observations, it was evident that higher concentrations of VOCs were required to be added to the artificial system (Supplementary material Table S1) compared to original headspace concentrations (Table 2). This indicates antagonists may produce these compounds in very high levels whereas we measure the amounts in headspace which are in an equilibrium after absorption into the growth medium.

In conclusion, a novel extraction setup was designed for automated extraction of VOCs by headspace-SPME-GC-MS for quantification of volatiles, when both antagonist and pathogen are in an interaction. The proposed system allows the introduction of an IS, without losing compounds. A total of fourteen VOCs were identified from *A. pullulans* by untargeted metabolomics followed by MCR-ALS. These included seven alcohols, three ketones and four esters. PCA and PLS-DA were further employed to discriminate between the volatile profiles of antagonist inoculated and non-inoculated samples. Four VOCs were identified as the most important features in discriminating samples based on the SR and VIP scores. Ethanol and 2-phenylethanol were found as key inhibitory *A. pullulans* VOCs for both *B. cinerea* and *A. alternata*. RSM optimised a cocktail of ethanol, 3-methyl-1-butanol, 2-methyl-1-propanol and 2-phenylethanol for the first time to minimise colony growth of *B. cinerea* and *A. alternata in vitro*.

## Supplementary information

Supplementary material.

## Data Availability

All relevant datasets generated during and/or analysed during the current study are promptly available from the corresponding author upon request.
