# Supplementary material for: Aureobasidium pullulans volatilome identified by a novel, quantitative approach employing SPME-GC-MS, suppressed Botrytis cinerea and Alternaria alternata in vitro
Source: Sci Rep. 2020 Mar 11;10:4498. doi: 10.1038/s41598-020-61471-8 (PMC7066187; doi:10.1038/s41598-020-61471-8)
Supplement: Supplementary file 1 — Supplementary material. [file 41598_2020_61471_MOESM1_ESM.pdf]

SUPPLEMENTARY MATERIAL FOR

Aureobasidium pullulans **volatilome identified by a novel, quantitative approach employing SPME-GC-MS, suppressed** ***Botrytis cinerea*** **and** ***Alternaria alternata in vitro***

S.M. Yalage Don*, L.M. Schmidtke, J.M. Gambetta, C.C. Steel

School of Agricultural and Wine Sciences, National Wine and Grape Industry Centre, Charles Sturt University, Locked Bag 588, Wagga Wagga, New South Wales 2678, Australia

* Corresponding author: S.M. Yalage Don

Address: School of Agricultural and Wine Sciences, National Wine and Grape Industry Centre, Charles Sturt University, Locked Bag 588, Wagga Wagga, New South Wales 2678, Australia

Email: syalagedon@csu.edu.au

Tel: +61 468 960 806

Table S1: Levels of concentrations of VOCs used in the Box-Behnken design and RSM for *A. alternata* and *B. cinerea*

**VOC**	**Levels of concentration (mg/L)**		
	**Low (-1)**	**Mid (0)**	**High (+1)**
Ethanol (*A. alternata* T1)	75,000	200,000	325,000
Ethanol (*B. cinerea)*	50,000	125,000	200,000
3-Methyl-1-butanol	750	2000	3250
2-Methyl-1-propanol	750	2000	3250
2-Phenylethanol	750	2000	3250

Table S2: ANOVA and significant values associated with coefficients for RSM

**Source**	**Model 1 (equation (2))**		**Model 2 (equation (3))**		**Model 3 (equation (4))**	
	Coefficients of regression	P-value	Coefficients of regression	P-value	Coefficients of regression	P-value
**Model**	-	< 0.0001		< 0.0001		< 0.0001
**Intercept**	+20.07	-	+34.73	-	+38.05	-
**A (Ethanol)**	-4.46	< 0.0001	-12.25	< 0.0001	-13.10	< 0.0001
**B (3-Methyl-1-butanol)**	-0.31	0.1795	-1.92	0.0087	-1.06	0.4809
**C (2-Methyl-1-propanol)**	+0.19	0.4141	-1.63	0.0232	-1.77	0.2450
**D (2-Phenylethanol)**	-0.58	0.0170	-2.63	0.0007	-6.56	0.0002
**B-D**	+0.94	0.0255	-	-	-	-
**A-D**	-	-	-	-	-6.00	0.0293
**Lack of fit**	-	0.2467	-	0.1854	-	0.1005

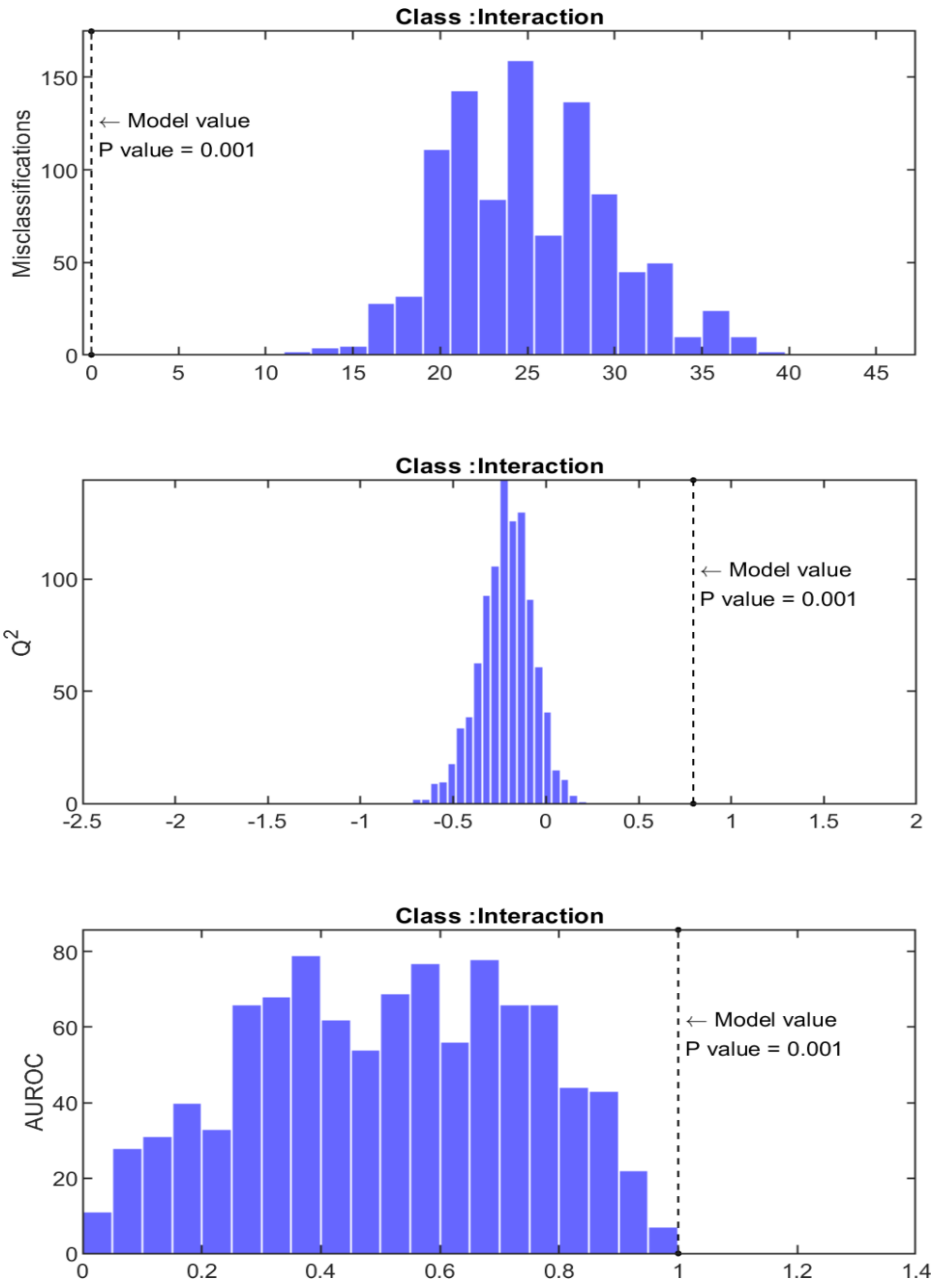

Figure S1. PLS-DA model validation for GC-MS data assigned to class interaction. Results based on cross model validation predictions of original labelling (vertical dashed line) compared to permuted data assessed using metrics of true model performance (Q^2, AUROC and number of misclassifications). Empirical p-values determined from 1000 permutations of data.

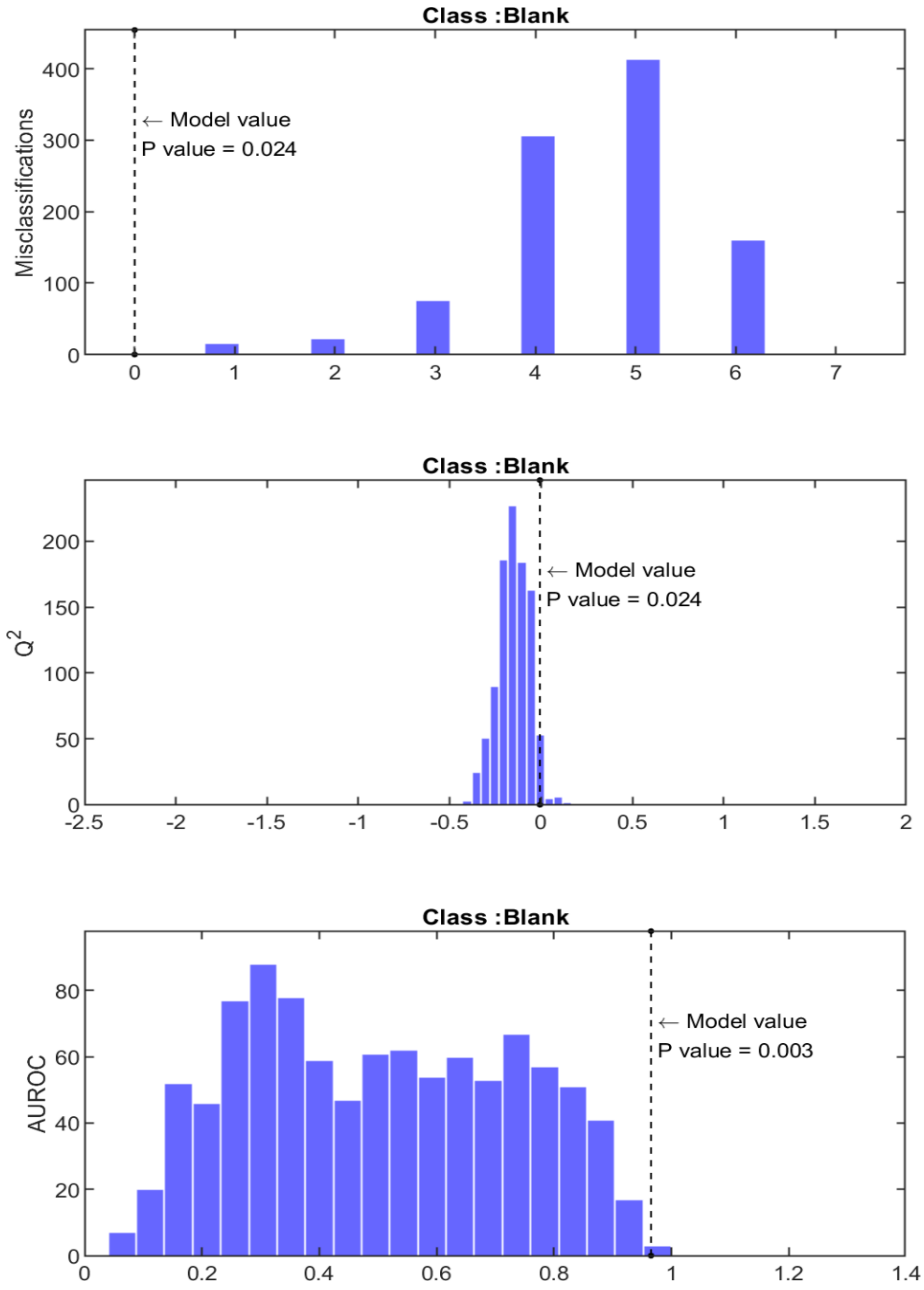

Figure S2: PLS-DA model validation for GC-MS data assigned to class blank. Results based on cross model validation predictions of original labelling (vertical dashed line) compared to permuted data assessed using metrics of true model performance (Q^2, AUROC and number of misclassifications). Empirical p-values determined from 1000 permutations of data.

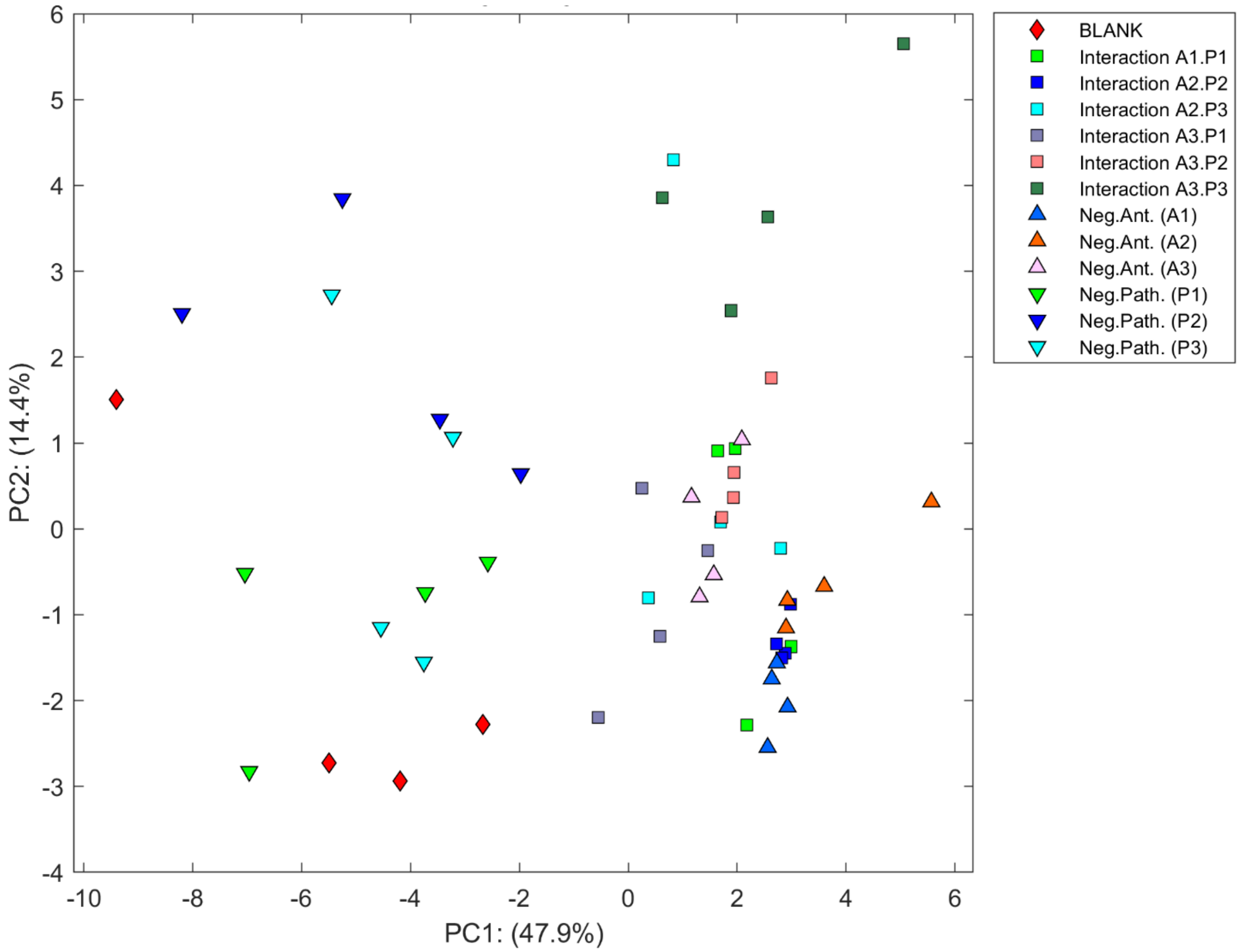

Figure S3: PCA scores plot coded for the samples of specific antagonist/pathogen combinations based on GC-MS normalized peaks areas, showing PC1 vs. PC2. Three *A. pululans* isolates (A1, A2 and A3) as negative pathogen, three pathogen isolates (*B. cinerea* TN080 (P1), *B. cinerea* DAR 69764 (P2) and *A. alternata* (P3)) as negative pathogen, PDA media blank and multiple interactions between *A. pullulans* and pathogens (A1-P1, A3-P1, A2-P2, A3-P2, A2-P3 and A3-P3) are presented each with four replicates.

(a)

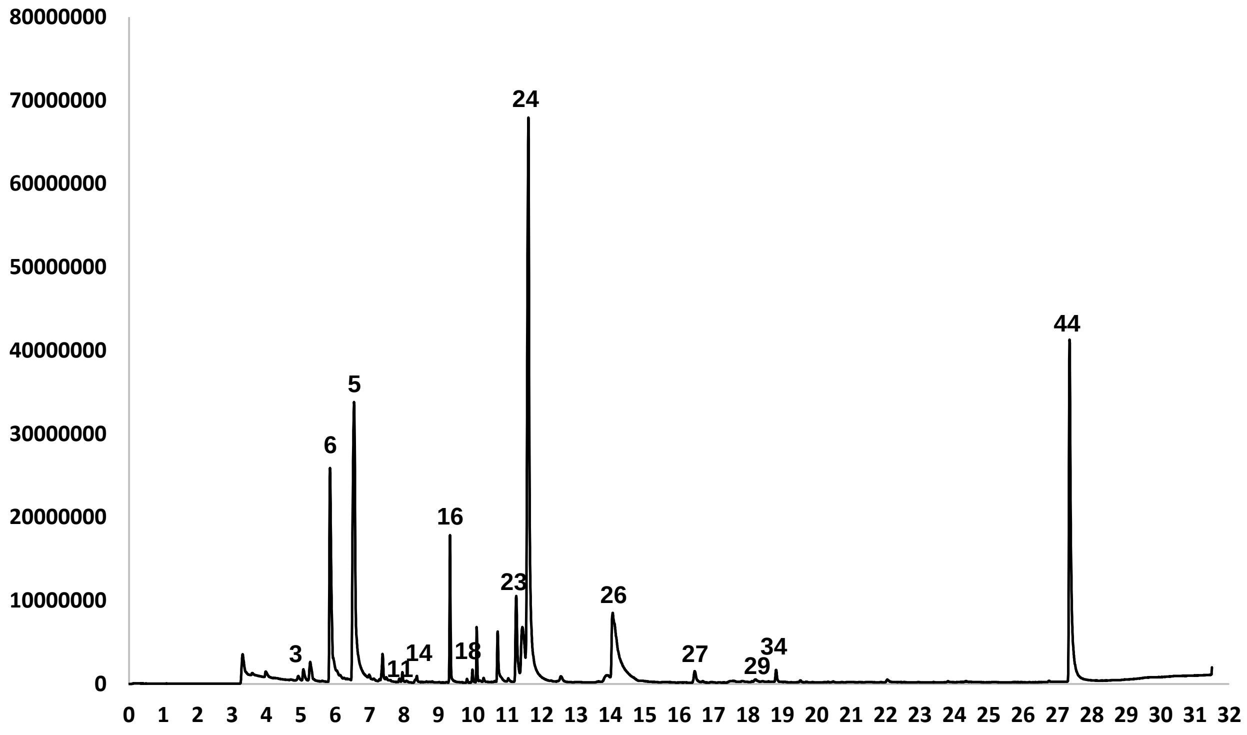
80000000
70000000
60000000
50000000
40000000
30000000
20000000
10000000
0
0 1 2 3 4 5 6 7 8 9 10 11 12 13 14 15 16 17 18 19 20 21 22 23 24 25 26 27 28 29 30 31 32
24
44
5
6
16
23
26
3
11
14
18
27
29
34

(b)

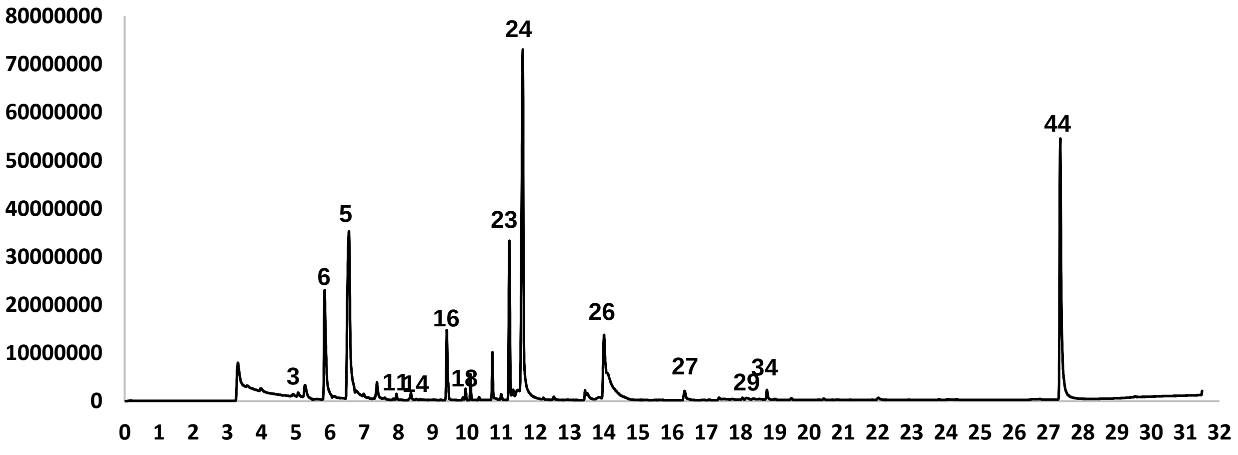
80000000
70000000
60000000
50000000
40000000
30000000
20000000
10000000
0
0 1 2 3 4 5 6 7 8 9 10 11 12 13 14 15 16 17 18 19 20 21 22 23 24 25 26 27 28 29 30 31 32
24
44
5
23
6
16
26
3
11
14
18
27
29
34

(c)

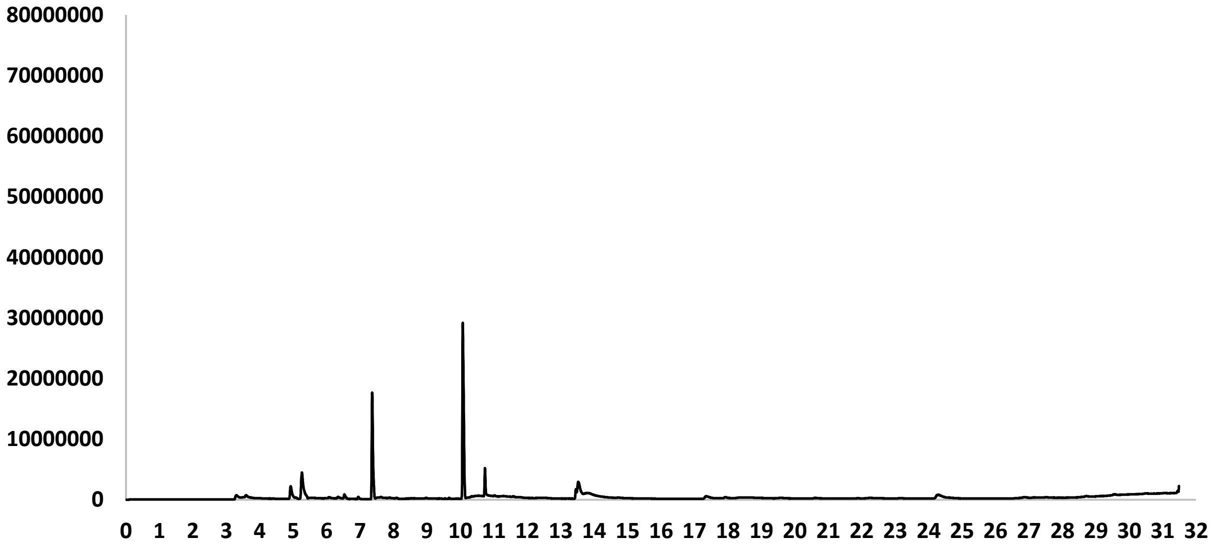

(d)

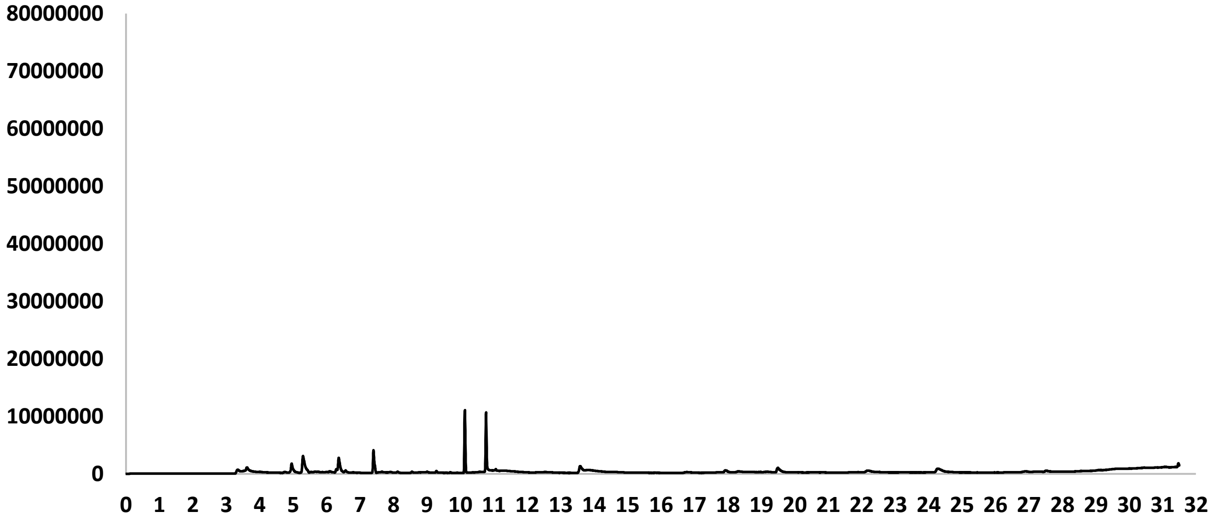

Figure S4: Representative chromatograms of the four types of samples, (a) negative antagonist (*A. pullulans* A2), (b) interaction (*A. pullulans* A2+*B. cinerea* DAR 69764, (c) negative pathogen (*B. cinerea* DAR 69764) and (d) media blank from the SPME-GC-MS untargeted compounds identification.

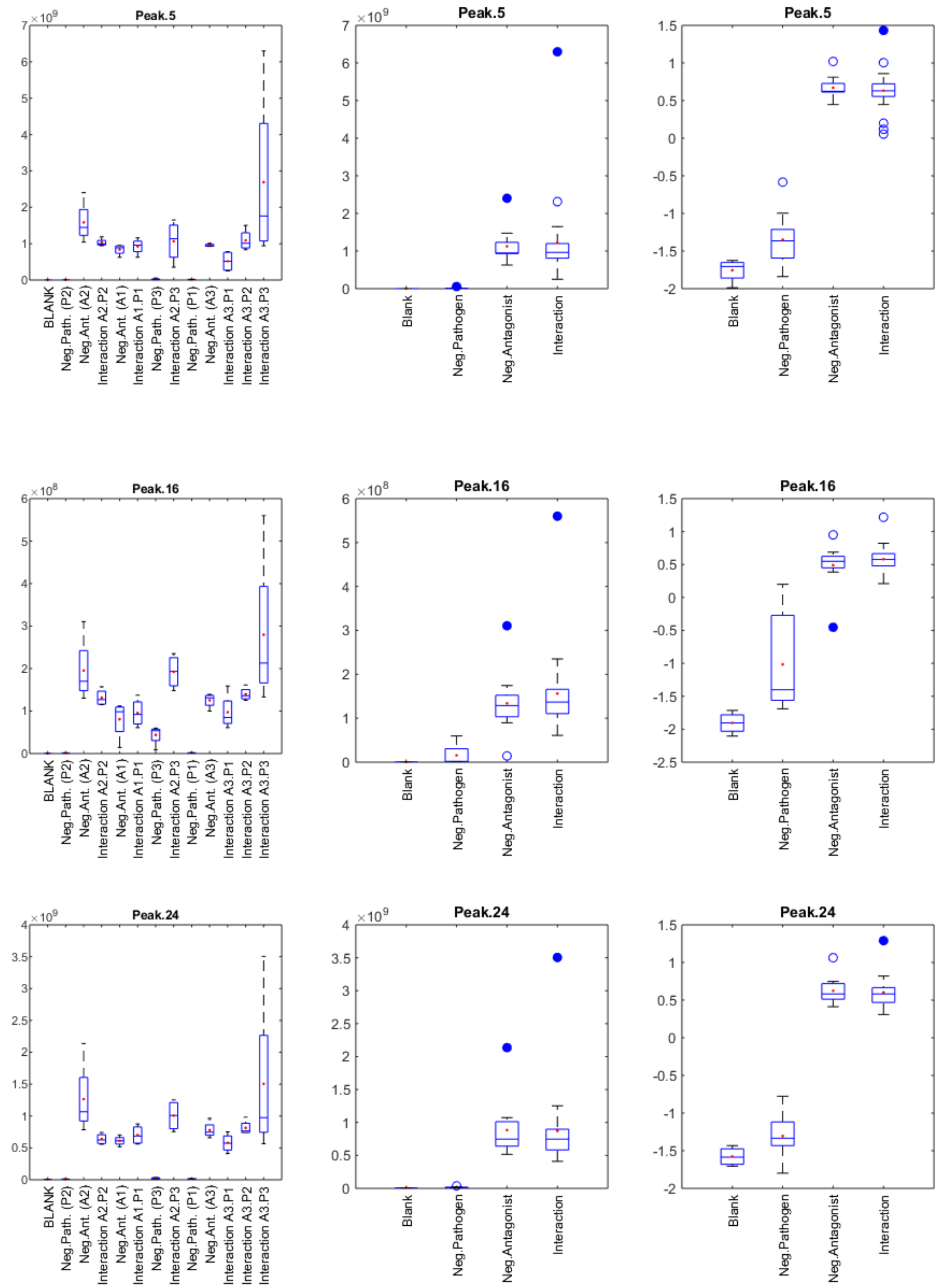
Peak.5
×10^9
BLANK
Neg.Path. (P2)
Neg.Ant. (A2)
Interaction A2.P2
Neg.Ant. (A1)
Interaction A1.P1
Neg.Path. (P3)
Interaction A2.P3
Neg.Path. (P1)
Neg.Ant. (A3)
Interaction A3.P1
Interaction A3.P2
Interaction A3.P3
Peak.5
×10^9
Blank
Neg.Pathogen
Neg.Antagonist
Interaction
Peak.5
Blank
Neg.Pathogen
Neg.Antagonist
Interaction
Peak.16
×10^8
BLANK
Neg.Path. (P2)
Neg.Ant. (A2)
Interaction A2.P2
Neg.Ant. (A1)
Interaction A1.P1
Neg.Path. (P3)
Interaction A2.P3
Neg.Path. (P1)
Neg.Ant. (A3)
Interaction A3.P1
Interaction A3.P2
Interaction A3.P3
Peak.16
×10^8
Blank
Neg.Pathogen
Neg.Antagonist
Interaction
Peak.16
Blank
Neg.Pathogen
Neg.Antagonist
Interaction
Peak.24
×10^9
BLANK
Neg.Path. (P2)
Neg.Ant. (A2)
Interaction A2.P2
Neg.Ant. (A1)
Interaction A1.P1
Neg.Path. (P3)
Interaction A2.P3
Neg.Path. (P1)
Neg.Ant. (A3)
Interaction A3.P1
Interaction A3.P2
Interaction A3.P3
Peak.24
×10^9
Blank
Neg.Pathogen
Neg.Antagonist
Interaction
Peak.24
Blank
Neg.Pathogen
Neg.Antagonist
Interaction

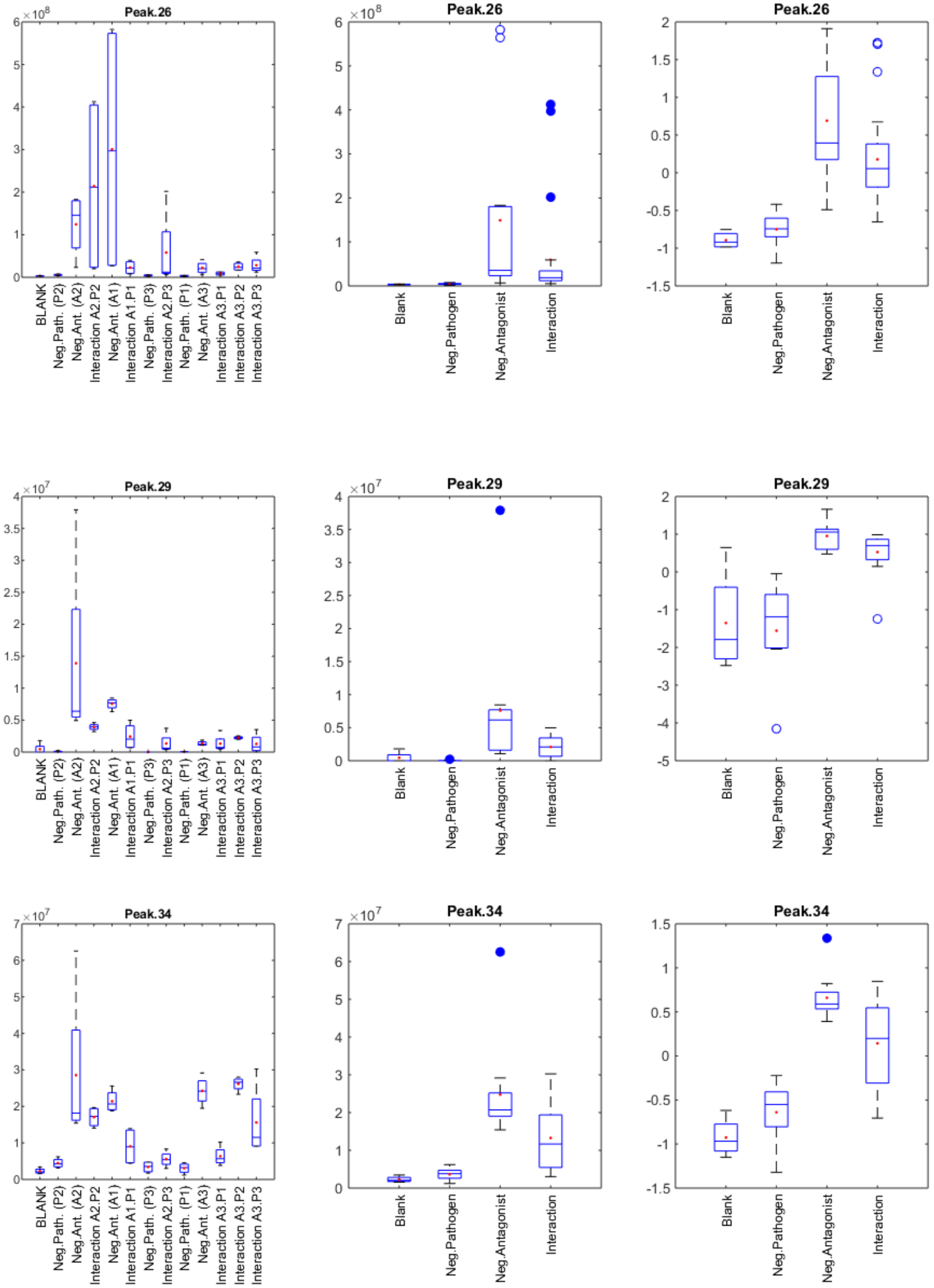
Peak.26
Peak.26
Peak.26
Peak.29
Peak.29
Peak.29
Peak.34
Peak.34
Peak.34
BLANK
Neg.Path. (P2)
Neg.Ant. (A2)
Interaction A2.P2
Neg.Ant. (A1)
Interaction A1.P1
Neg.Path. (P3)
Interaction A2.P3
Neg.Path. (P1)
Neg.Ant. (A3)
Interaction A3.P1
Interaction A3.P2
Interaction A3.P3
Blank
Neg.Pathogen
Neg.Antagonist
Interaction

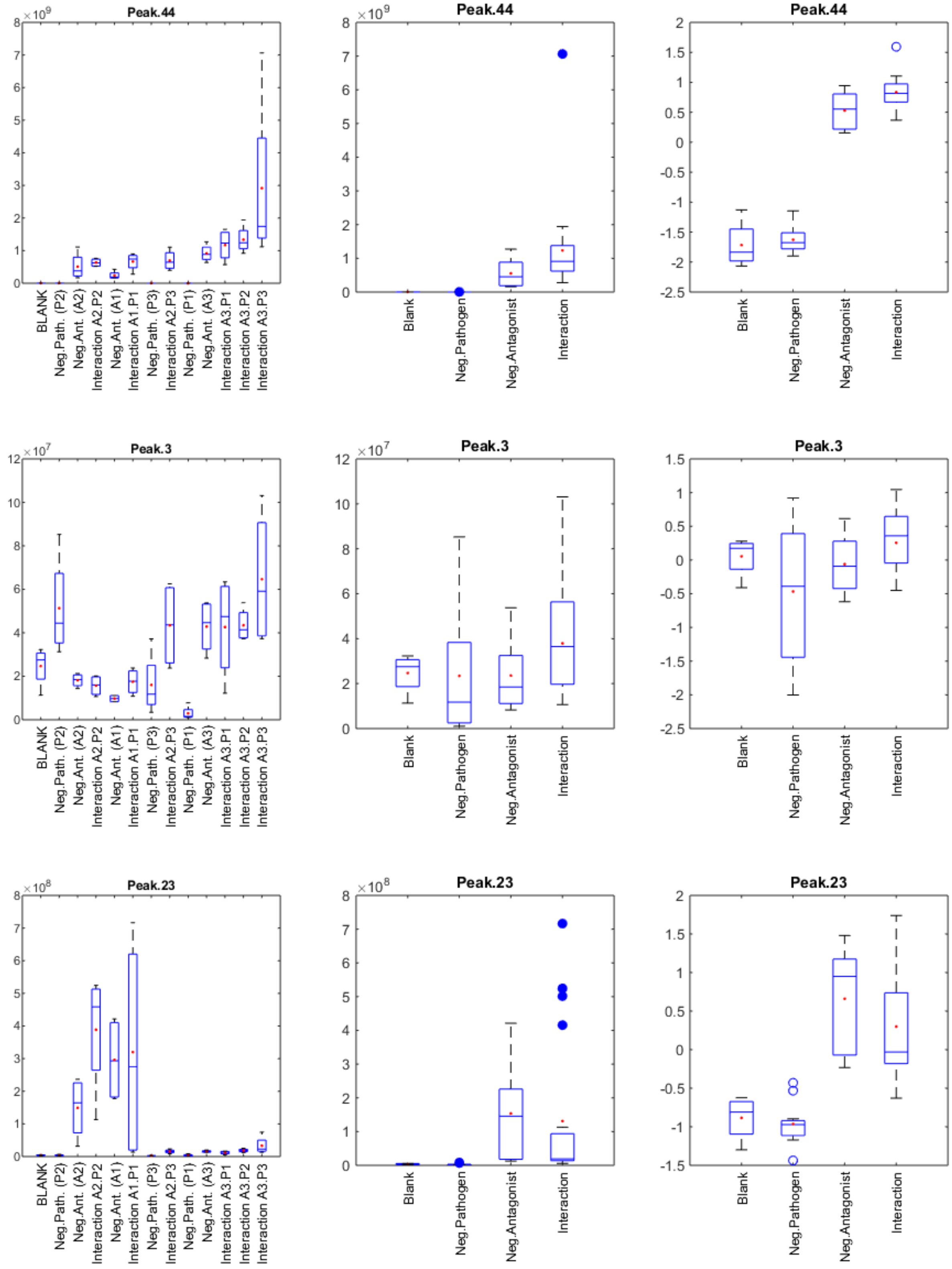
Peak.44
×10^9
BLANK
Neg.Path. (P2)
Neg.Ant. (A2)
Interaction A2.P2
Neg.Ant. (A1)
Interaction A1.P1
Neg.Path. (P3)
Interaction A2.P3
Neg.Path. (P1)
Neg.Ant. (A3)
Interaction A3.P1
Interaction A3.P2
Interaction A3.P3
Peak.44
×10^9
Blank
Neg.Pathogen
Neg.Antagonist
Interaction
Peak.44
Blank
Neg.Pathogen
Neg.Antagonist
Interaction
Peak.3
×10^7
Peak.3
×10^7
Peak.3
Peak.23
×10^8
Peak.23
×10^8
Peak.23

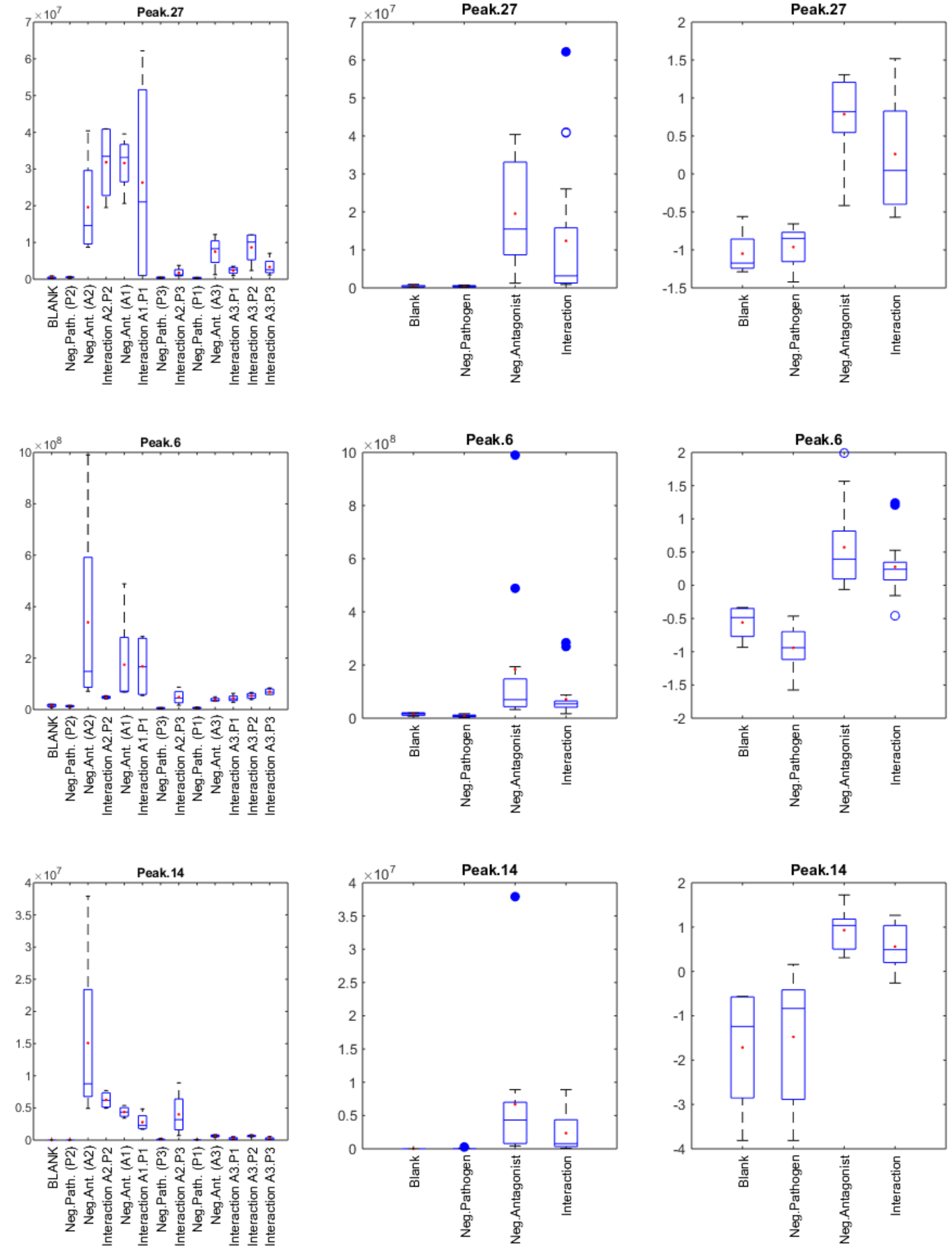
Peak.27
Peak.27
Peak.27
Peak.6
Peak.6
Peak.6
Peak.14
Peak.14
Peak.14
BLANK
Neg.Path. (P2)
Neg.Ant. (A2)
Interaction A2.P2
Neg.Ant. (A1)
Interaction A1.P1
Neg.Path. (P3)
Interaction A2.P3
Neg.Path. (P1)
Neg.Ant. (A3)
Interaction A3.P1
Interaction A3.P2
Interaction A3.P3
Blank
Neg.Pathogen
Neg.Antagonist
Interaction

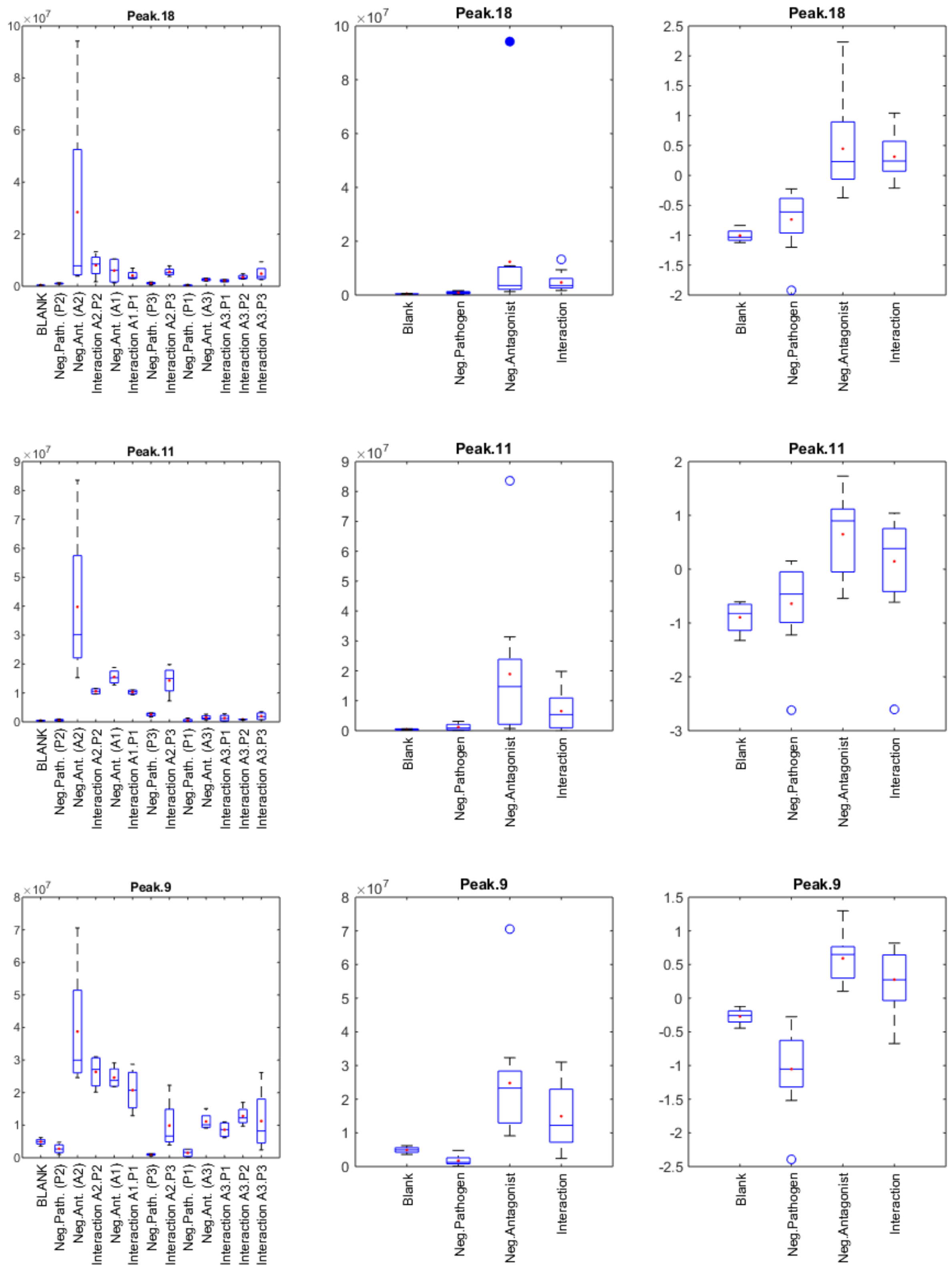
Peak.18
Peak.11
Peak.9
×10^7
BLANK
Neg.Path. (P2)
Neg.Ant. (A2)
Interaction A2.P2
Neg.Ant. (A1)
Interaction A1.P1
Neg.Path. (P3)
Interaction A2.P3
Neg.Path. (P1)
Neg.Ant. (A3)
Interaction A3.P1
Interaction A3.P2
Interaction A3.P3
Blank
Neg.Pathogen
Neg.Antagonist
Interaction

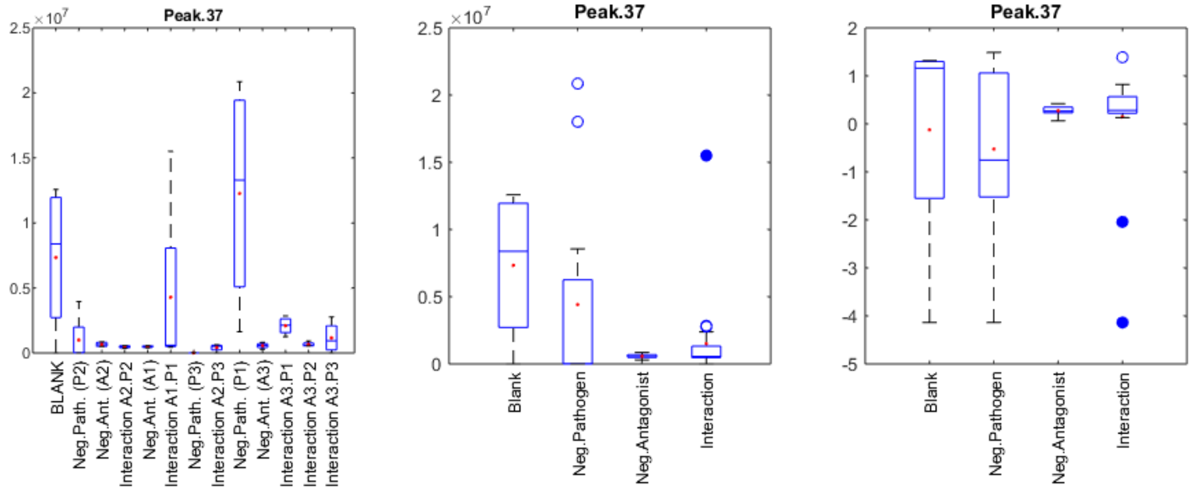

Figure S5: Box-plots of features abundances normalized to the IS. These include plots of fifteen compounds from *A. pullulans* culture headspace and compound #37 which seem to have a greater influence on sample discrimination in PCA scores plot. In every set of three plots for each peak, plot 1 is abundances for samples, plot 2 shows the abundances of sample groups and plot 3 is the grouped samples with features which are mean centred, logarithmic and variance scaled using the Pareto approach.
